# Functional and Material Properties in Nanocatalyst Design: A Data Handling and Sharing Problem

**DOI:** 10.3390/ijms22105176

**Published:** 2021-05-13

**Authors:** Daniel Lach, Uladzislau Zhdan, Adam Smolinski, Jaroslaw Polanski

**Affiliations:** 1 Institute of Chemistry, Faculty of Science and Technology, University of Silesia, Szkolna 9, 40-006 Katowice, Poland; daniel.lach@us.edu.pl (D.L.); uladzislau.zhdan@us.edu.pl (U.Z.); 2 Central Mining Institute, Plac Gwarkow 1, 40-166 Katowice, Poland; asmolinski@gig.eu

**Keywords:** catalyst property prediction, catalysts informatics, infrastructure for catalyst property prediction, data sharing in catalyst discovery, data handling in catalyst discovery, cheminformatics for material discovery, catalytic material database, data science, data collection

## Abstract

(1) Background: Properties and descriptors are two forms of molecular in silico representations. Properties can be further divided into functional, e.g., catalyst or drug activity, and material, e.g., X-ray crystal data. Millions of real measured functional property records are available for drugs or drug candidates in online databases. In contrast, there is not a single database that registers a real conversion, TON or TOF data for catalysts. All of the data are molecular descriptors or material properties, which are mainly of a calculation origin. (2) Results: Here, we explain the reason for this. We reviewed the data handling and sharing problems in the design and discovery of catalyst candidates particularly, material informatics and catalyst design, structural coding, data collection and validation, infrastructure for catalyst design and the online databases for catalyst design. (3) Conclusions: Material design requires a property prediction step. This can only be achieved based on the registered real property measurement. In reality, in catalyst design and discovery, we can observe either a severe functional property deficit or even property famine.

## 1. Introduction

Chemical compounds can be represented by molecular descriptors or properties. Essentially, drug or material design requires that these representations be mapped in order to predict the properties of the targeted materials quantitatively or qualitatively. Descriptors can be calculated from a molecular representation, but properties can only be measured, most often for substances [1,2]. Accordingly, the availability of the measured property data is crucial in material design. Moreover, material, particularly catalyst design, is an example of the early identification of a forward and inverse design problem. The forward problem relates “the chemical composition and/or high-level descriptors of the composition to the performance of the material in the application of interest.” In contrast, in the inverse one, we relate the performance to the desired chemical to composition or formulation. Formally, design is the solution to the inverse problem [3]. In other words, in a forward mode, we are mapping molecular descriptors to properties while in the inverse one—properties to descriptors [4].

A lack of property data is a well-known bottleneck in drug design. The broadening range of properties that are available in material (drug) design will enable better predictions of the functionalities targeted. This, in turn, would enable more perspective products to be developed. The first problem is the fact that taking property measurements is expensive. Therefore, we need novel methods that would make property measurements easier and more efficient. The latest idea of the lipidomics in drug design is a clear illustration. In lipidomics, the range of the lipids that are released after a drug is administered is registered. We then have a two-dimensional fingerprint and not a single property value that codes the behavior of the substance [5]. However, taking physical measurements is only one problem. The availability of the property data that is measured is at least of the same importance. For example, we need to know of any negative results in order to avoid failures, and these results are not usually published. Accordingly, data sharing is a critical problem that challenges drug and material discovery. The databases that register the properties have steadily gained in their importance, and therefore collaborative discovery could be a more advanced solution that would enable the drug (material) candidate data to be collected or substance libraries to be assembled. Open-source drug discovery is an example [6]. The second problem is that, surprisingly, property, predicted property and descriptor typology are not only ill-defined for complex materials, but also for chemical compounds. Molecular weight (MW), which can be either a measured property, a predicted property or molecular descriptor, is a good example. Even the term chemical compound ambiguously refers to both the molecules or substances in experiments or a virtual in silico environment [1,2].

A variety of novel ideas appeared recently in materials discovery. The materials genome initiative (MGI) is an example of a project evidently inspired by drug design. MGI connects the high throughput method (HTM: for the importance of HTM in catalysts discovery compare reference [7]) with theoretical simulations, e.g., DFT calculations with experimental and open-source computer tools. Currently, the time between discovery and commercialization of advanced materials is 10–20 years. The aim of MGI is to cut the time needed for materials commercialization to 5–10 years and to lower commercialization costs [8]. In this article, we review the range of properties that can be measured when designing novel catalysts that are available in the databases. Properties can be divided into functional, e.g., catalyst or drug activity, and material, e.g., X-ray crystal data. Millions of real measured functional property records are available for drugs or drug candidates in online databases. In contrast, not a single database registers a real conversion for catalysts, i.e., TON or TOF data. All of the data are molecular descriptors or material properties, mainly of a calculation origin. Here, we explain the reason for this. Moreover, we ask the question of collaborative discovery in this area. We review the data handling and sharing problems in the design and discovery of catalyst candidates, particularly material informatics and catalyst design, structural coding, data collection and validation, infrastructure for catalyst design and online databases for catalyst design. This is because we have recently been investigating methanation catalysts [9,10,11,12], this review is specifically focused on nanocatalysts for methanation.

## 2. Property Production in Drug and Catalyst Design

**Figure 1:**
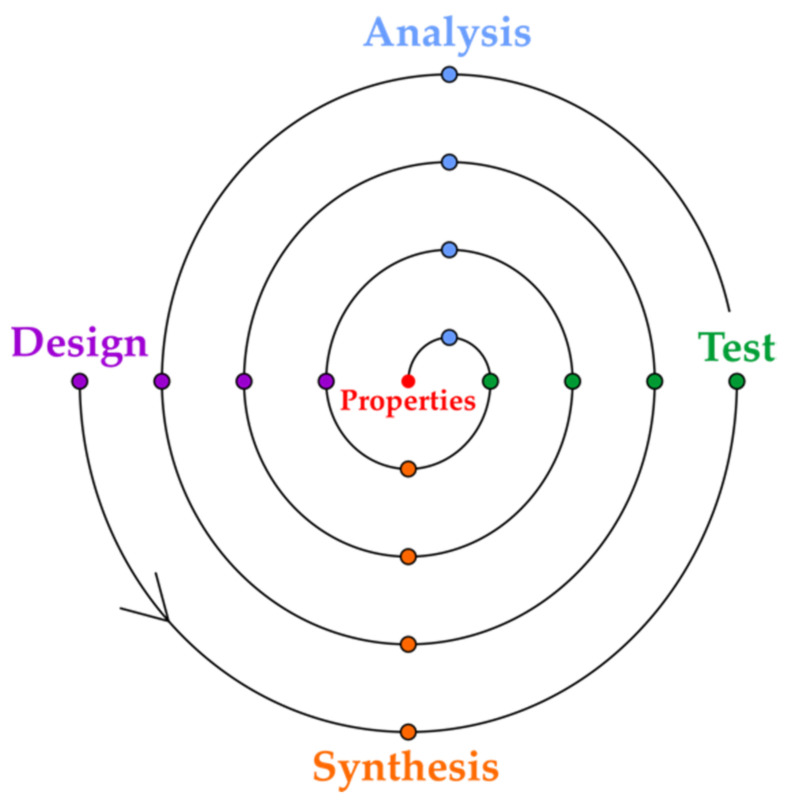
The spiral of the optimization of the property design cycles in material or drug discovery.

Chemists focus on the construction of a variety of functional materials that are used as drugs, preservatives, flavors, etc. On average, a single new compound makes nothing among the millions of registered chemical compounds in reverse to a compound of a desired property. While chemical syntheses are often complex, property design or prediction is far more complicated and is still lacking efficiency. In practice, novel compounds are often synthesized or chemical systems are constructed in the quest to find the property in a trial and error strategy. This effect was already described by Hammond, who coined the term property production [13]. A paradox of chemistry is that property production and design, and not the synthesis design, is what creates the real interest. Usually, property production requires an optimization that involves iterative steps of design, synthesis, testing and analysis (Figure 1).

**Figure 2:**
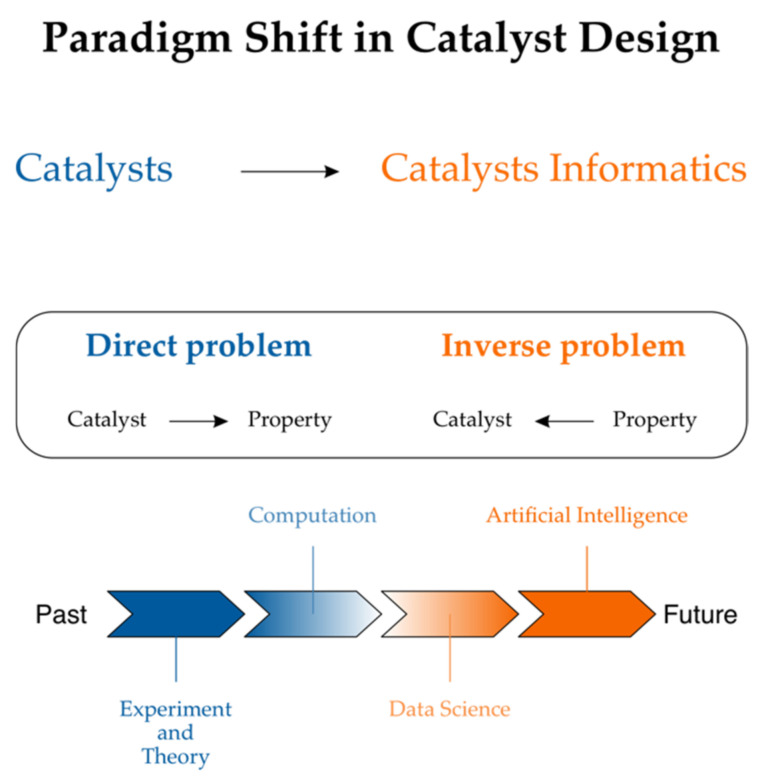
Catalyst informatics for property production follows cheminformatics in drug design. Reprinted with permission from [22]. Copyright © 2021 John Wiley and Sons.

An unprecedented improvement in small molecule synthesis and synthesis design has been observed recently [14,15,16,17]. What about property design? The importance of designing novel drugs in silico led to the field of chemoinformatics [18] in order to address solely this issue at its origin [4]. However, Eroom’s law, which states that drug discovery becomes slower and more expensive over time despite improvements in technology, illuminates the complications that have been encountered. As a result, the best-selling drugs are getting older. The lack of innovation can be illustrated, for example, by the fact that only a few new antibiotics are being discovered [19,20]. If the contrast between synthesis vs. property design in drug designs is rationalized, we must understand that the drug moiety is only a small part of the complex construction of the drug-receptor structure. System chemistry is a new branch of chemistry that attempts to move beyond the reductionism of studying multi-component molecular objects. In this branch of chemistry, drug functionality is to be designed as an integral part of a complex chemical system whose construction resembles a material more than a small organic molecule. Actually, in the current literature, drugs are sometimes designated as materials. Generally, the evolution from chemical compounds to materials is what was observed in recent years. With the development of new analytical methods, chemists have the potential to manipulate and combine chemical elements into structures that are more complex than chemical compounds. Although materials are this type of structure, Molecular Organic Light-Emitting Diodes (OLED) are examples in which materials are simply chemical compounds. The recent success of OLED material and property design is worth noting [21]. In this context, the parallel between drugs and materials is even closer. Materials chemistry is based on advanced physical and chemical characterization. In turn, until just recently, material design was addressed less seriously than drug design. Material informatics, which follows cheminformatics, is a relatively recent concept and catalyst genomics was evidently inspired by the increasing importance of genomics in drug design [22]. Figure 2 illustrates a recent concept of catalyst informatics.

**Figure 3:**
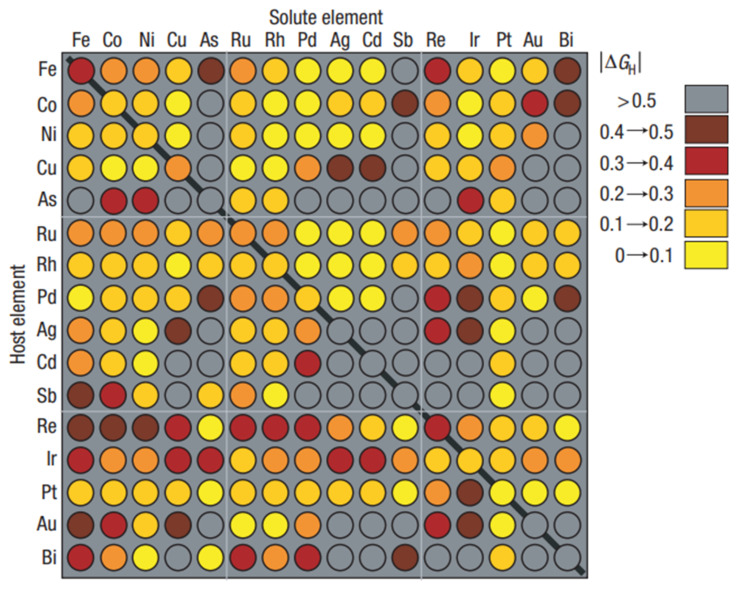
Simulation matrix for the HTS screening of metal and metal alloys. The ∆G_H,_ which was calculated in silico is coded by the colors. The rows indicate the pure metal values, while the indicate the bimetallic solutes that were embedded in the individual metal surface layers. Reprinted with permission from [30]. Copyright © 2021 Royal Society of Chemistry.

With the increasing role of materials informatics, the importance of rational simulations and data handling has also increased [22,23,24,25,26]. For example, the need for a more precise description of the performance relationship of the catalyst structure with the efficiency that is at least similar to that of drug design has recently been realized [27]. The concept of catalyst informatics follows the concept of drug design, e.g., the so-called direct and reverse structure–property (activity) problem appeared. In drug design, properties are usually designed by predicating their structural features and not their properties directly. Then, by comparing the properties as a function of structural changes, the required properties can be designed. This method is known as indirect property design. In the direct method, the property is designed directly. Using the direct method is still quite rare and is still a concept more than a reality, even in drug design. However, paradoxically, the direct methods could be more easily available in materials (catalysts) informatics than in drug design, because the interactions of catalysts with the environment are less complex than those of the drugs interacting with the biological environments. The first-principle theoretical simulations should probably be interpreted as the direct design [28]. A high-throughput material screening is an example in which a direct theoretical evaluation of a material property is performed in silico [29,30]. The catalyst activity of binary surface alloys was simulated in silico to find electrocatalytic materials for hydrogen evolution. Figure 3 presents the results [30].

Takahashi et al. identified three important concepts in catalyst informatics, which are catalyst data, catalyst data to catalyst design and the platform for catalyst informatics [22]. Specifically, catalyst informatics involves data handling, including the literature data, as well as the analysis and validation of these data. The property–performance relationships for efficient CO_2_ hydrogenation into higher hydrocarbons over Fe-based catalysts is an example of this type of research, in which the authors not only conducted a literature query but also performed an experimental validation of the previous results [31]. Synergistic material combinations are important elements in catalyst design. We recently indicated how the privileged structure concept can be used to find a privileged metal combination in bimetallic nanocatalysis [10]. One experimental approach in this area could be the design of the library of the potential catalysts based on the literature of oxidative methane coupling, in order to test the reliable relationships between the performance of catalysts and the synergistic combinations within the broad material combinations [32]. Data mining supported by artificial intelligence was used in the search for catalysts for the low-temperature oxidative coupling of methane [33].

**Figure 4:**
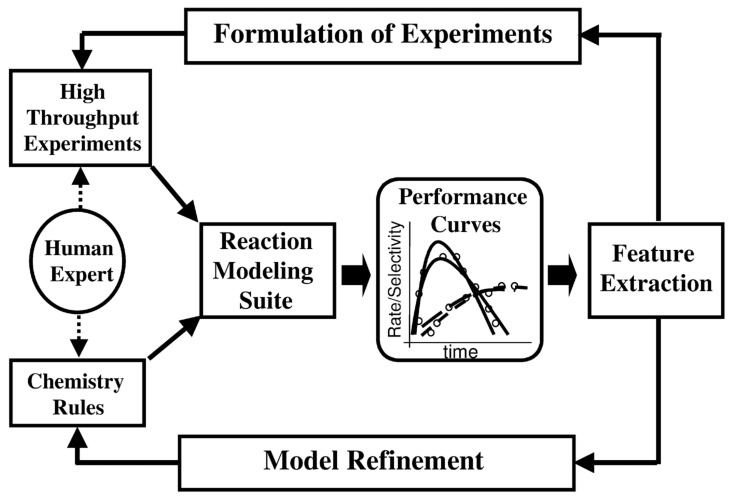
Knowledge extraction in the HTS catalyst forward design. Reprinted with permission from [3]. Copyright © 2021 Elsevier.

In practice, the high-throughput screening approaches appeared highly successful, both in drug and catalysis design. Interestingly, this method can be classified as a complex property to the structure mapping approach or design mode, where a property of a single chemical compound or a material system is expanded to a multinumber variable. Caruthers et al. explain how the catalyst HTS modeling framework can be supported by the “*knowledge extraction (KE) engine transparently mapping rules-to-equations-to-parameters-to-features as part of the forward model*.” Figure 4 is a schematic illustration for such design mode [3]. In drug design, where we search for the leading structures among promising chemical compounds, the current HTS methods can include the screening of the commercial compounds library of a size of 10^5^ to find a low activity substance, of which the structure can be then improved to shape a drug candidate. Usually, robots are supporting the search at this stage. An early example of HTS is the Creer et al. approach, which screened zeolites as the potential catalysts of cyclopropane conversion [34]. Other HTS experiments in this area were also described [35,36,37].

## 3. From Data to Catalyst: In Silico Material Representations for Mapping the Properties of Catalyst Candidates

**Figure 5:**
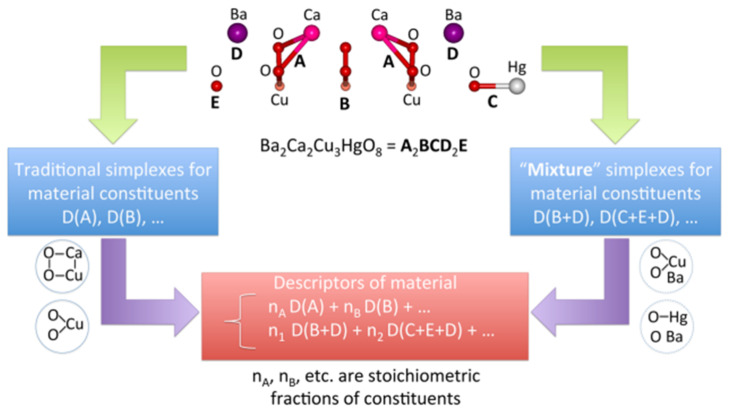
The simplex method for generating the SiRMS descriptors for materials. Reprinted with permission from [39]. Copyright © 2021 American Chemical Society.

The drugs that are designed and being tested are called drug candidates. We can use a typology that is similar to materials. Molecular descriptors, i.e., the parameters that code molecular structures, are an essential counterpart of the measured properties for predicting the properties using the indirect property design method. A variety of multidimensional descriptors were developed for describing potential drugs [38]. In turn, catalysts, which are often more complex than chemical compounds, are much more complicated to describe. The so-called materials cartography method [39] has recently been suggested for coding such structures as fragmental codes in the form of a Simplex representation of the molecular structure (SiRMS; Figure 5) [40,41].

An example of the materials cartography helps to understand the structure representation problem in silico. However, different nanostructures or active surfaces, and not bulk material structures, are of critical importance for the catalytic performance. The SiRMS coding can even be extended to a direct in silico representation by the Cartesian coordinates of atomic positions, which could code the whole structural information. A variety of such methods were described for chemical compounds where Cartesian coordinates could also be transformed to Kohonen maps of atomic positions [42], or molecular surfaces [43,44] or molecular autocorrelation vector [45], which allows for mapping various structure to properties. Similar in silico representations were also described for catalysts, e.g., the Cartesian coordinates of atomic positions were used for coding nanoclusters in the distance-based machine learning methods. Similar to chemical compound data, the Cartesian coordinates needed a pre-transformation to be invariant to translation, rotation or permutation. The transformation of the Cartesian coordinates of atomic positions to many-body tensor representation appeared successful for DFT simulations of Au_38_(SCH_3_)_24_ nanoclusters [46].

**Figure 6:**
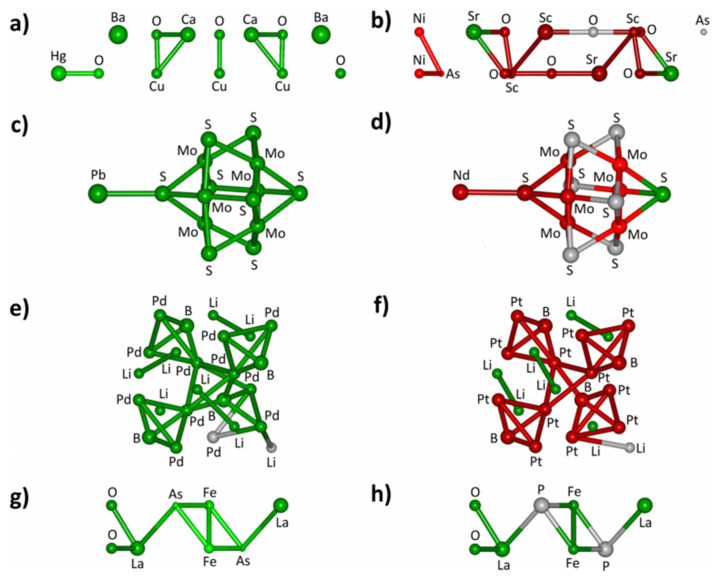
SiRMS representation of the material fragments that are influential and not-influential to a functional material property. Structural fragments that decrease the superconductivity critical temperatures (CT) are colored in red and those that enhance CT are shown in green. Non-influential fragments are in gray. (**a**) Ba_2_Ca_2_Cu_3_HgO_8_; (**b**) As_2_Ni_2_O_6_Sc_2_Sr_4_; (**c**) Mo_6_PbS_8_; (**d**) Mo_6_NdS_8_; (**e**) Li_2_Pd_3_B; (**f**) Li_2_Pt_3_B; (**g**) FeLaAsO and (**h**) FeLaPO. Reprinted with permission from [39]. Copyright © 2021 American Chemical Society

The SiRMS coding enables the structures that contribute to a certain functional property or activity to be identified (Figure 6).

QSAR is a method that is designed to predict a property in drug design. The QSAR method can be applied directly to catalysis when catalysts are individual chemical compounds. For example, the 3D QSARs of this type in single-site polymerization catalysts were reviewed in publication [47]. DFT and QSAR studies of ethylene polymerization by zirconocene catalysts are other studies of this type [48]. Computational ligand descriptors for catalyst design were reviewed in publication [49].

We should remember, however, that the current function of classical QSAR in drug design is limited. In particular, in multidimensional QSAR [50], we usually relate a massive number of molecular descriptor data to a single property while the number of active molecules is low. This unbalance decides the predictivity of the models and is problematic. The MI-QSAR aimed at predicting and modeling molecular permeability through the cell membrane is an example of such a model [51]. Currently, by QSAR, we often mean the models with a significantly larger number of objects where QSAR can be coupled with the enormously massive databases of factual and/or virtual molecular objects [52,53]. Property production is a substantial target of chemical science. In this sense, Butler et al. differentiated chemical science into molecular and materials sciences [52]. More precisely, we should discuss chemical compounds or substances constructed directly by individual compounds, species or more complex chemical systems.

Typically, materials are much more complex than chemical compounds; therefore, molecular QSAR needs modifications. The Quantitative Materials Structure–Property Relationship (QMSPR) is the QSAR equivalent in materials informatics [50]. Here, we are expecting the analysis of much more complex structural information than simple chemical compounds.

## 4. Data Sharing in Drug and Catalyst Design: From Catalyst Candidates to Commercial Catalysts

The best drug or material candidates, e.g., catalyst candidates, are expected to win as innovations in the market. While new ideas are important for successful innovation in material engineering, ideas for boosting creativity are also necessary. In a way that is similar to natural evolution meeting and mating, ideas lead to new ideas and innovation. The sophistication of modern technology is not due to individual knowledge and skills but rather to collaboration and collective enterprise [54]. This idea gained popularity for drug design, where the appearance of collaborative drug design (CCD) projects was recently observed. Knowledge and data sharing, data curation, or even creating chemical compound libraries, are the main cooperation schemes within these projects. Data must not only be collected and shared, but it is also necessary to correct any errors in the reported information. Otherwise, the designed materials or drugs would not be rationally functional. This operation is known as data curation. Curating the protein information in the Protein Data Bank (PDB) is an illustrative example of this procedure [55]. Collaborative Drug Design (CDD) is a modern research informatics platform that supports collaborative drug discovery, which helps project teams manage, analyze and present data for biotech companies, contract research organizations (CRO), academic labs, research hospitals, agrochemical and consumer goods companies. Knowledge sharing is another goal of the platform. A series of webinars are available that discuss the drug design issues that are usually hidden in big pharma laboratories. The examples can be demystifying machine learning (artificial intelligence) or the Chris Lipinski vs. Burry Bunin discussion on the controversies of the Lipinski Rule of 5, which is a benchmark for contemporary drug design [56]. The European Lead Factory is another example of a collaboration platform that not only focuses on data sharing and curation, but that formed the Joint European Compound Library (JECL), which collected more than 321,000 compounds from the proprietary collections of seven pharmaceutical companies in 2013–2018 and will collect as many as 500,000 compounds that will be available for collaborative testing in different projects [57,58]. The shared platform for antibiotic research and knowledge (SPARK) is a specific collaborative tool to spark antibiotic discovery [59]. An open-source drug design is a synonym of the above-mentioned CDD strategies [60].

**Figure 7:**
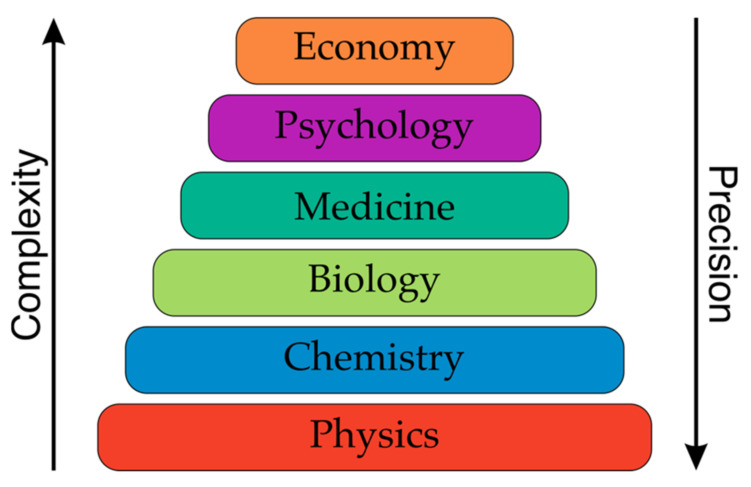
Complexity of the sciences from physics to the economy. Inspired from [1].

In drug design, the actual activity (functional property) of drug candidates, as well as the chemical compounds that are being investigated, are collections of huge data repositories, e.g., the PubChem or CHEMBL databases where 2,435,467 or 779,714 numerical potency records are available. Interestingly, a significant amount of these data contrasts with ca. 2000 substances in registered drugs. In turn, the functional properties, e.g., TON, TOF, for catalysts is quite scarce. The excuse for this is a fact that conversion, selectivity, TON and TOF strongly depend on the reaction conditions and the environment. It should, however, be remembered that there are similar problems in medicinal chemistry, where assays provide highly artefactual results [61]. Thus, what could be the reason that medicinal chemistry pragmatically registers the functional property data and catalyst chemistry does not. An answer can be found in Figure 7, which describes the complexity of the sciences. The lower the level of the science, the more precise the description. Catalyst discovery and design can be placed somewhere between physics and chemistry, where a high level of precision is expected. However, in medicinal and biological chemistry, which involves chemistry and biology, a much higher level of uncertainty is accepted. Therefore, it is quite clear that the biological activity of the chemical compounds that are measured in different labs are usually different. The use of reference substances is one solution to this problem.

The need for data sharing and collaboration has also been noticed in material research. Projects such as the Integrated Collaborative Environment (ICE) [62], or the Materials Genome Initiative (MGI) [63,64], are good examples of this. The goal of ICEs is to create a cyberinfrastructure that accelerates material innovation through the seamless flow and connection of information. The MGI strives to strengthen the collaboration between multiple institutes by focusing on the integration of experiments, computation and theory throughout the material development cycle. The computer modeling of materials is, in turn, supported by the Materials Cloud [65,66]. This platform enables open and seamless resource sharing and archiving and hosts modeling services, analytical and pre-/post-processing tools and educational materials. In addition, the recorded data are citable. Because most modern materials are nanomaterials, NanoHUB should also be mentioned. NanoHUB is a web-based interface that brings together the people working in materials nanotechnology and provides them with interactive simulation and modeling tools and educational materials, as well as a platform for the exchange and dissemination of research data [67,68]. The initiative to create professional networks, joint projects and a content and application management system was extended to many other disciplines by HUBzero [69,70]. A similar infrastructure is AiiDA [71,72], which is based on the Python code and is mainly dedicated to computational research. Because another group of special interest is catalysts, the Catalyst Acquisition by Data Science (CADS) [73] should also be mentioned. This platform enables the sharing and publishing of catalytic data, as well as their visualization, analysis and exploration [74]. It uses prediction and analysis tools that are based on machine learning and provides a space for cooperation.

## 5. In Silico Design of Heterogenous Catalysts

Data mining and the big data concept plays a role in material discovery in the past few years. For any given organic compound, the synthetic connections between millions of chemical substances that are associated with billions of synthetic possibilities recorded in the massive chemical information repositories can be explored in seconds. Data mining is the convenient or automated extraction of the data patterns that represent knowledge from the apparently unstructured data that are implicitly captured and stored in large databases. During the data mining process, machine learning techniques are required.

Data mining is a multistep procedure. First, the data are collected, preprocessed and normalized. Next, machine learning algorithms are trained and tested to acquire meaningful data, analyze the processed information and represent it in a standardized format. Finally, the data mining progression results are used to predict any significant features. Data mining is primarily used to test a hypothesis or to discover some new or hidden patterns [75]. A detailed review of the above-mentioned problems is available in reference [52]. This includes such problems as reclaiming the literature by natural language processing to identify information from the unstructured text sources. ChemDataExtractor is an example of a toolkit for the automated extraction of chemical information from the scientific literature. Such systems can be used to process the plain text and also figures and tables integrated within the text. The software converts various input formats into a universal record that consists of a single linear stream of elements (paragraphs and tables are each processed independently). This utility is especially important for materials science, where a lot of data are reported as figures, e.g., catalyst conversion vs. temperature performance. The extracted information is merged into a specific collection of chemical records. Interestingly, the ChemDataExtractor is available as an open-source python package from http://www.chemdataextractor.org (accessed on 22 March 2021) and can be used for free. An overview of the complete information extraction system, as well as the illustrative examples of its use, can be found in reference [76].

### 5.1. Data Science

Data science concepts and artificial intelligence began a new digital age in the fields of catalysis [77,78], chemistry [25,79] and materials science [52,80,81]. The parameters that are collected via data mining can be used to teach machine learning algorithms in order to predict numerous values of activity, selectivity and to define the degree of excellence of a material as a catalyst for a specific reaction. In this way, catalyst preparation has evolved from trial-and-error methodologies, which are based on chemical knowledge, and accumulated experience and common sense into a clearly multidisciplinary science. The frontiers of machine learning for materials science were reviewed in reference [52]. In particular, Butler et al. indicated the increasing predictivity of the computational chemistry offered in the XXI century by machine learning and artificial intelligence accelerating the design, synthesis, characterization and application of materials. The routine application of computer models can broaden this area to experimental chemists, or even non-specialists. Machine learning usually needs large datasets, but there are efforts to extract *more knowledge from smaller datasets*. Efficient chemical in silico representations, quantum learning, automatic discovery of scientific laws, and principles are other important discovery areas in machine learning that would contribute to materials discovery [24,52,78]. Some more specific problems can appear while processing a large number of data, e.g., if one normalizes the data prior to machine learning and estimates the performance of the model using cross-validation, it may result in an overly optimistic model, which is known as the so-called “data leakage” (compare: https://machinelearningmastery.com/data-leakage-machine-learning/, accessed on 5 May 2021).

The development of surface-characterization techniques, molecular modeling and advanced synthesis methods have changed the preparation of solid catalysts into an art of science. Heterogeneous catalysis lies at the intersection between materials science, chemistry and physics, and it instantly gains all of the benefits from developments in those fields. Furthermore, heterogeneous catalysis is a multi-scale phenomenon to which machine learning can be applied to improve the various levels of theories that are used to describe the effects that arise at different time and length scales. For example, machine learning was used to design new materials [39,82,83], predict molecular properties [84,85,86], reduce the cost of simulating chemical systems [87,88,89,90,91], improve the accuracy of quantum methods [92,93,94] and develop efficient force fields [83].

The first task for machine learning is to identify the descriptors that can be used to predict and understand the target properties from raw data. While the descriptors can be any synthesis method or physical property based on the structure of the material [95], the targets are usually selectivity, activity and stability.

The machine learning process first starts with a set of questions to which it should find an answer. Next, it continues through data acquisition, a data transmutation procedure to make it accessible to computer algorithms, and then it trains an explanatory or predictive model on the obtained data and evaluates the final result.

One of the main tasks is to create a database in which the data will be machine-readable, accessible and easily discoverable by humans. This can be achieved using the standard protocols (such as https request), thereby ensuring simple, secure and unified user access. Application Programming Interfaces (API) and human-friendly web interfaces are the ones to obtain this user access. Many Databases, such as HTEMDB (High Throughput Experimental Materials Database) or the Materials Project, use standard solutions such as data fetching over http, which ensures the interchangeability of a queue to different databases. It is worth noting that the Python libraries, such as Python Material Genomics, highly support data access for the analysis of materials. Using the Resource Description Framework (RDF) for data transfer makes the data–metadata relationship machine-readable. This data should be described with enough details to reobtain the result so that it can be used in another context.

**Figure 8:**
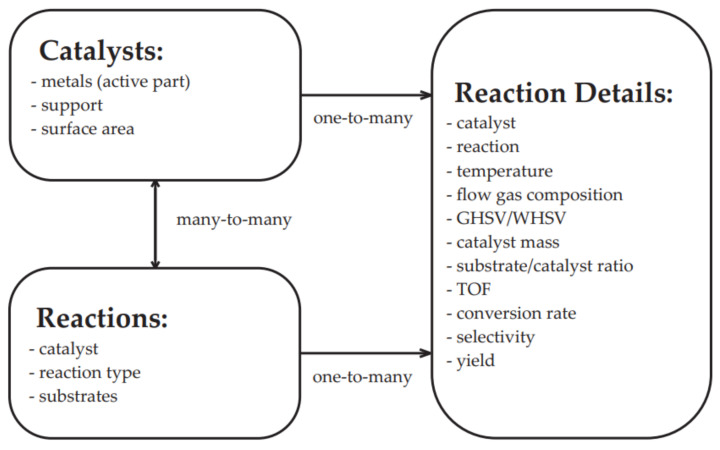
Schematic of the data structure of a Catalytic Material Database SQL.

Relational databases, such as MySQL and SQLite are generally better suited for storing well-defined collections of properties. The data are arranged in ordered tables with columns and rows that can be linked to capture the connections between different types of entries. The power of relational databases is the ability to select subsets of data with a high degree of efficiency by constraining the value of one or more of the columns. An illustration of the SQL data structure is presented in Figure 8.

Fingerprinting is a method that is used to represent a material with regard to descriptor features. Incidentally, features in data science can refer both to molecular descriptors and properties. The subsets of relevant features, which can themselves be used as simple surrogate models for the target, are molecular representations of the descriptor type [96]. The features should preferably have a physical meaning and be easily accessible. Additionally, they should be universal, which makes them valid, strong predictors across a variety of different catalyst types. Ideally, it should be possible to infer the catalyst material from a given set of promising features, which can be seen as the reverse process [80] of featurization. SIRMS (Figure 5 and Figure 6) are an example of descriptors that could be used as a physical code fingerprinting catalyst structure.

**Figure 9:**
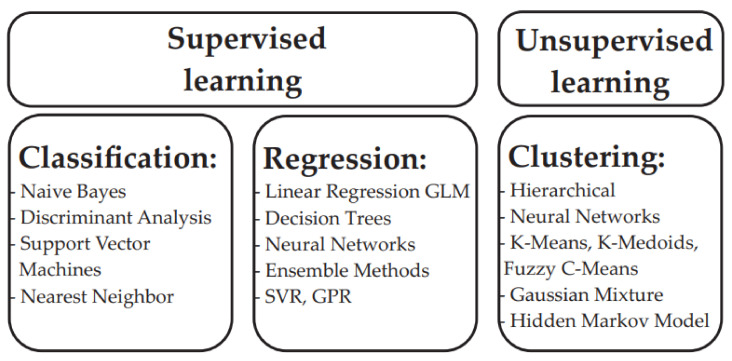
Machine learning methods.

Machine learning models ideally start by selecting a target problem and collecting critical data. Then, the obtained data are described, modeled and/or used for in silico simulations. Depending on the target, some algorithms are more suitable than others. Machine learning models that are trained on atomic/material data are typically good for interpolating with similar systems but have less precision extrapolating with other materials, which is a general phenomenon in ML. For this reason, one desirable property of a machine learning model is the ability to universalize the predictions to data that are different from the training set. Different types of ML methods are presented in Figure 9.

### 5.2. Machine Learning Methods

The most common form of machine learning is supervised learning. In supervised learning models, the ML algorithm maps a set of features to a huge set of training data, thus enabling the model to examine the output and to adjust parameters until the desired results are obtained [75]. The primary supervised learning tasks usually include regression, prediction, or classification.

Unsupervised learning methods enable a machine to explore a set of unlabeled data. After the initial exploration, the machine tries to identify any hidden patterns that may help to establish theories and that can be used to facilitate later predictive studies. These algorithms turn the data into groups that are based only on statistical variables. Unsupervised models do not require training on substantial data sets and therefore they are much easier and faster to deploy than the supervised learning methods.

### 5.3. Deep Learning

An important subfield of machine learning is deep learning. The quintessential deep learning models are the deep feedforward neural networks, which use multiple layers of artificial neurons. These models are called feedforward because the information flows through the layers of neurons without any feedback connections. The inclusion of feedback connections creates recurrent neural networks [97]. Deep learning can be seen as an extreme case of model stacking. Besides the basic feedforward hidden layer architecture, we can add layers that have diverse functionalities, such as pooling layers and normalization layers, which are used in convolutional neural networks. Therefore, the earlier layers in deep (convolutional) networks can be interpreted as automatic feature extractors. Specifically, deep neural networks were seen as black-box models because of the lack of weight interpretability.

Deep learning networks and neural networks are powerful for interactions between non-linear features. The performance of neural networks typically increases with the amount of training data in situations in which other machine learning models have already reached an asymptotic performance level [97]. Furthermore, NN architecture is of crucial importance for the computational efficiency of a system. The disadvantages are the complexity of their architectures and their difficult implementation. Depending on the model, neural networks may need a lot of training data to obtain the appropriate results. Furthermore, although neural networks lack any intuitive interpretability, their analysis methods are constantly improving.

### 5.4. Integrating Synthesis with Machine Learning

How can the synthesis of the desired structures be realized and integrated into machine learning? We usually need here much larger data; therefore, more and more often, robots are replacing chemists. This can be observed for chemical compounds [98] or materials [99]. However, a question is how general these methods will appear. A more traditional approach was described by Gómez-Bombarelli et al., who screened 1.6 million virtual molecules using DFT to identify novel OLED targets. The best candidates were selected and synthesized [21].

## 6. Data for Catalyst Design

Catalysts for heterogeneous catalysis are often complex objects. They consist of metal combinations, supports and often intermediate layers or promoters. Combining the properties of various materials enables the boundaries that are imposed by the ubiquitous single-component volcano relationship to be exceeded [100]. The scaling relationships for adsorption and the energy of the reaction transition state are then not constrained and the optimal tuning of catalyst activity or selectivity in subsequent catalytic sequences becomes possible [100,101,102]. The materials can be combined to provide better efficiency, e.g., to higher reaction rate by using one material to lower the dissociation barrier and another to lower the reaction barrier. These materials are not limited by the same adsorbate scaling set and Brønsted–Evans–Polanyi (BEP [103,104]) relationship [105,106]. Otherwise, it would not be possible to obtain an overall activity that exceeds that of an optimal mono-material catalyst.

On the other hand, it is also crucial to use the appropriate experimental design methods [107]. The goal here is to eliminate any variables that have little impact on performance, quantify the relationships between the variables and responses, and perform a sensitivity analysis. For example, for systematically changing catalyzed reaction conditions, a response surface model can be calculated in which the parameters such as temperature, pressure, GHSV and stoichiometry are related to the responses, i.e., conversion, yield and selectivity. Such models are quite useful for creating the optimal conditions for optimal performance. However, the search for catalytic materials with the target properties must be described by both the data, fundamental (descriptors or predicted properties) and empirical (measured properties). Therefore, it is important to collect this data in an orderly manner and to include the possibility of reorganizing and exporting it to any format so that its processing is both easy and widely available. In this context, Table 1 shows a list of the selected databases that are available for materials design in heterogenous catalysis.

The data stored in these databases are primarily the predicted data and data that are related to the material properties. To the best of our knowledge, there are no databases that record the functional properties of catalytic materials yet. In addition, there is only a small amount of material data that were measured experimentally. Therefore, the design of catalysts requires that specific functional properties be obtained under given conditions. Parameters such as conversion and selectivity under a constant reaction condition, turnover frequency (TOF), turnover number (TON), space velocity for a given or constant conversion and space-time yield (STY) are critical [108]. For instance, TOF enables the number of turnovers of the catalytic cycle per unit of time to be determined and TON is used to determine the number of maximum uses of a catalyst [108,109]. In turn, STY shows the quantity of the desired product per catalyst volume in the unit time. These data are necessary for performing comparative measurements, determining the process parameters or conducting catalyst deactivation tests. The availability of this type of data is necessary and that is why a database of functional property database for methanation, hydrogenation and deNOx catalysts, as discussed in Section 4, was created.

Table 1 is a list of the selected databases that are available for materials design in heterogeneous catalysis. According to the materials project websites, some of the calculated parameters available are not reliable and deviate largely from the experimental value, e.g., the band gap. This could be the issue to develop other databases as well. Data-sharing projects could help to solve this issue.

**Table 1:** Selected databases for the design of catalytic materials.

Database Vs. Data	Inorganic Materials Database (AtomWork)	Materials Project	High Throughput Experimental Materials Database (HTEM DB)	The Open Quantum Materials Database (OQMD)	Computational 2D Materials Database (C2DB)	CatApp Database	Catalysis Hub	ChemCatBio Catalyst Property Databases (CPD)
Crystallographic and structural	✓	✓	-	✓	✓	-	-	-
Thermal and thermodynamic or kinetic	✓	✓	-	✓	✓	✓	✓	✓
Electronic and electrical	✓	✓	✓	✓	✓	-	-	-
Mechanical or magnetic	✓	-	-	-	✓	-	-	-
Optical	✓	-	✓	-	✓	-	-	-
Phase diagrams	✓	✓	-	✓	-	-	-	-
XRD ^1^, XRF ^2^, XAS ^3^	✓ - -	✓ - ✓	✓ ✓ -	- - -	- - -	- - -	- - -	- - -
Energy on the catalyst surface	-	-	-	-	-	✓	✓	✓
Available at: (Reference)	[110]	[111]	[112]	[113]	[114]	[115,116]	[117]	[118]

^1^ X-ray diffraction, ^2^ X-ray fluorescence, ^3^ X-ray absorption spectroscopy, (✓ - -) - XRD data, (✓ ✓ -) - XRD and XRF data, (✓ - ✓) - XRD and XAS data, (- - -) - no data.

## 7. The Database of the Functional Properties for Heterogeneous Nanocatalysts

**Figure 10:**
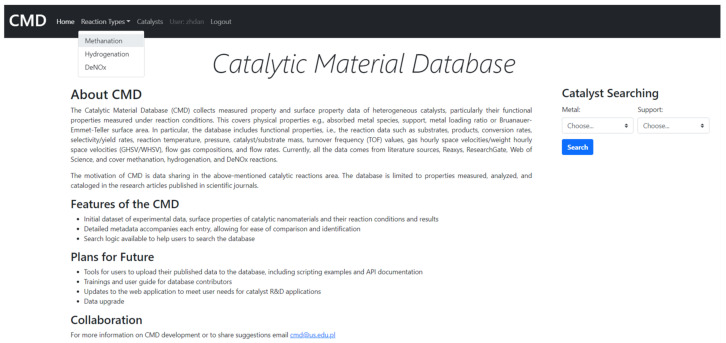
CMD home page.

The Catalytic Material Database (CMD) is a collection of experimental data and surface properties of heterogeneous catalysts and data about their reaction conditions. The data comes from primary literature sources and general databases: Reaxys, ResearchGate, the Web of Science, etc. The Catalytic Material Database website is available online: cmd.us.edu.pl, accessed on 12 April 2021. The appearance of the home page is shown in Figure 10.

**Figure 11:**
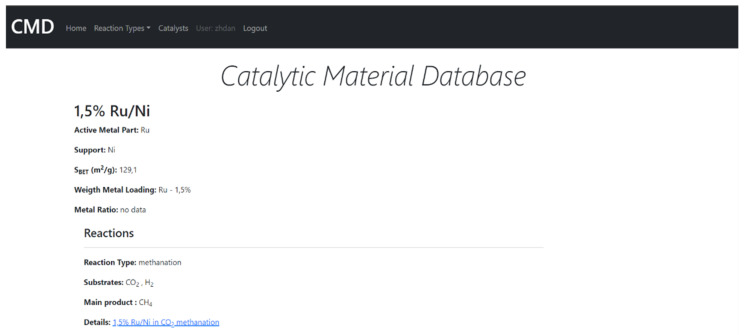
The example of the catalyst view mode of CMC.

**Figure 12:**
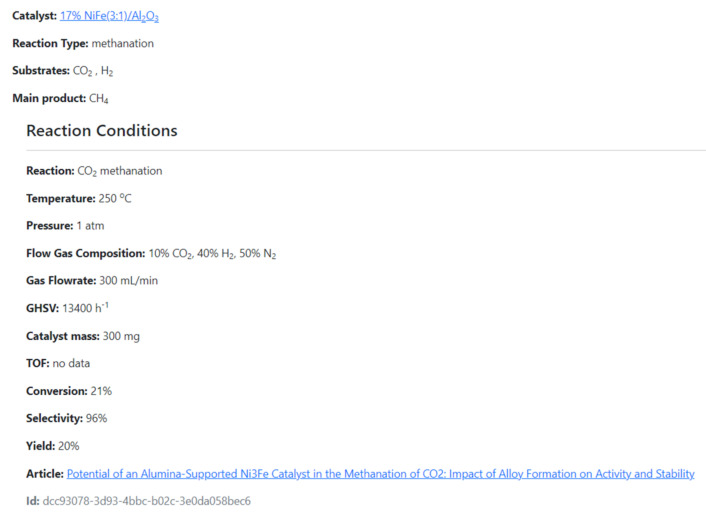
The example of the reaction view mode of CMC.

The CMD has a set of data for the physical properties of catalysts, e.g., absorbed metal species, support, metal loading ratio or the Bruanauer–Emmet–Teller surface area. Furthermore, the Database also includes functional properties, i.e., the reaction data such as substrates, conversion rates, products, selectivity/yield rates, reaction temperature, pressure, catalyst/substrate mass, turnover frequency (TOF) values, gas hourly space velocities/weight hourly space velocities (GHSV/WHSV), flow gas compositions and flow rates. The data covers mainly methanation. However, currently also hydrogenation, and DeNOx reactions (as environmental reductions) were included. The example of the data included in CMD is given in Supplementary Materials. The CMD website is designed for data sharing. A catalyst searching module is added. In Figure 11 and Figure 12, we show the examples of the catalyst and reaction view modes of the registered data.

**Figure 13:**
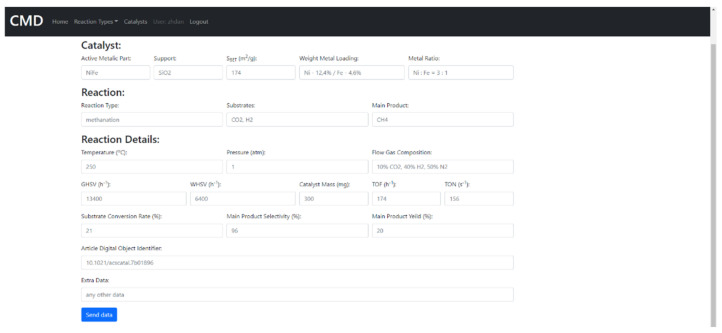
The data upload form in CMD.

The data upload by the database users is also possible (Figure 13).

## 8. Conclusions

Properties and descriptors are two forms of molecular in silico representations. Properties can be further divided into functional, e.g., catalyst or drug activity, and material, e.g., X-ray crystal data. Millions of actual measured functional property records are available for drugs or drug candidates in online databases. In contrast, not a single database registers the actual conversion, TON or TOF data for catalysts. All of the data are molecular descriptors or materials properties, which are mainly of a calculation origin. The reason for this is a lack of the reproducibility of the measurements in individual labs. Interestingly, similar problems in medicinal chemistry were pragmatically overcome by the use of reference substances. Generally, material design requires a property prediction step. This can only be achieved on the basis of the registered real property measurements. In reality, there is a severe functional property deficit, or even a property famine, in catalyst design and discovery. Data sharing is common in drug design. Accordingly, we reviewed data handling and data sharing problems in the design and discovery of catalyst candidates. Specifically, we examined materials informatics and catalyst design, structural coding, data collection and validation, infrastructure for catalyst design and online databases for catalyst design.

## Data Availability

Not applicable.

## Supplementary Materials

The following are available online at https://www.mdpi.com/article/10.3390/ijms22105176/s1, Table S1: CatData.xls.
